# Metagenomic shotgun sequencing reveals host species as an important driver of virome composition in mosquitoes

**DOI:** 10.1038/s41598-021-87122-0

**Published:** 2021-04-19

**Authors:** Panpim Thongsripong, James Angus Chandler, Pattamaporn Kittayapong, Bruce A. Wilcox, Durrell D. Kapan, Shannon N. Bennett

**Affiliations:** 1 Department of Microbiology, Institute for Biodiversity Science and Sustainability, California Academy of Sciences, San Francisco, CA USA; 2 Center of Excellence for Vectors and Vector-Borne Diseases, Faculty of Science, Mahidol University At Salaya, Nakhon Pathom, Thailand; 3 Global Health Group International, ASEAN Institute for Health Development, Mahidol University At Salaya, Nakhon Pathom, Thailand; 4 Department of Entomology and Center for Comparative Genomics, Institute for Biodiversity Sciences and Sustainability, California Academy of Sciences, San Francisco, CA USA; 5 Center for Conservation and Research Training, Pacific Biosciences Research Center, University of Hawai’i At Manoa, Honolulu, HI USA

**Keywords:** Microbiology, Virology, Metagenomics, Microbial ecology

## Abstract

High-throughput nucleic acid sequencing has greatly accelerated the discovery of viruses in the environment. Mosquitoes, because of their public health importance, are among those organisms whose viromes are being intensively characterized. Despite the deluge of sequence information, our understanding of the major drivers influencing the ecology of mosquito viromes remains limited. Using methods to increase the relative proportion of microbial RNA coupled with RNA-seq we characterize RNA viruses and other symbionts of three mosquito species collected along a rural to urban habitat gradient in Thailand. The full factorial study design allows us to explicitly investigate the relative importance of host species and habitat in structuring viral communities. We found that the pattern of virus presence was defined primarily by host species rather than by geographic locations or habitats. Our result suggests that insect-associated viruses display relatively narrow host ranges but are capable of spreading through a mosquito population at the geographical scale of our study. We also detected various single-celled and multicellular microorganisms such as bacteria, alveolates, fungi, and nematodes. Our study emphasizes the importance of including ecological information in viromic studies in order to gain further insights into viral ecology in systems where host specificity is driving both viral ecology and evolution.

## Introduction

Viruses are critically important to human and environmental health, and their diversity is predicted to be vast^1,2^. Next Generation Sequencing (NGS) technology has precipitated the discovery of many viruses, and expanded our knowledge of virus diversity, taxonomy, and evolution^3–5^. The RNA viruses of arthropod species, including mosquitoes, have been intensively characterized in the past several years, and stand out in their unparalleled diversity^6–19^.

Disease vectors such as mosquitoes pose a significant threat to public health. One sixth of the illness and disability suffered worldwide is due to vector-borne diseases, many of which are caused by mosquito-borne RNA viruses^20^. Examples of these viruses are dengue virus of the family *Flaviviridae*, chikungunya virus of the family *Togaviridae*, Rift Valley fever virus of the family *Phenuiviridae*, and Lacrosse virus of the family *Peribunyaviridae*. In addition to pathogenic viruses, myriad other mosquito-associated RNA viruses belonging to at least 15 other families do not pose direct public health concerns^21^.

Most virome studies apply NGS tools to identify new viruses and describe their diversity. This type of study may lead to the discovery of insect-specific viruses that have the ability to interfere with arbovirus transmission^22–26^. To maximize throughput while minimizing the sequencing cost, discovery-based studies have benefited from combining a large number of insects into a few pooled samples. Often, individuals from different species, or multiple locations, are pooled together hence information on their host-specificity and geographical location is lost^6,11,14^. The paucity of ecological information in virome studies limits our understanding of the host ranges of these viruses relative to their geographical distribution. As a result, we have little information on the relative importance of ecological drivers determining viral community structure.

In this study, we characterized RNA viruses and other microorganisms in pools of individuals of three common but overlooked vector species in Thailand: *Armigeres subalbatus*, *Culex fuscocephala*, and *Mansonia uniformis,* collected from three habitat types along a previously characterized rural to urban gradient^27^. Our full factorial design (all three mosquito species were collected at all three sites) allows us to explicitly determine the relative importance of host species and habitat in structuring viral communities. As obligate intracellular parasites, viruses rely on specific molecular interactions with hosts to perform their biological functions. Thus, we hypothesize that the pattern of virus presence in our mosquito samples is defined primarily by host species rather than by the host geographical locations or habitats. Finally, to maximize the sensitivity of NGS, we tested a laboratory method^28^ to deplete host ribosomal RNA, and increase the relative proportion of microbial genetic material prior to sequencing. This method could help reduce the need to pool samples across locations and species, and maintain ecological information in future virome studies.

## Methods

### Mosquito collection and RNA extraction

We described study site characteristics and adult mosquito collection in detail in a previous publication^27^. In summary, the study sites were located along a forest to urban landscape gradient in Nakhon Nayok province of central Thailand. Adult mosquitoes were collected during the rainy season of 2008 using a combination of trap types (BG Sentinel, Mosquito Magnet, CDC light trap, and CDC backpack aspirator). In total, over 83,000 adult mosquitoes were collected and transported to the laboratory on dry ice. Species identification based on available morphological keys revealed 109 species of female mosquitoes^29–34^.

**Figure 1:**
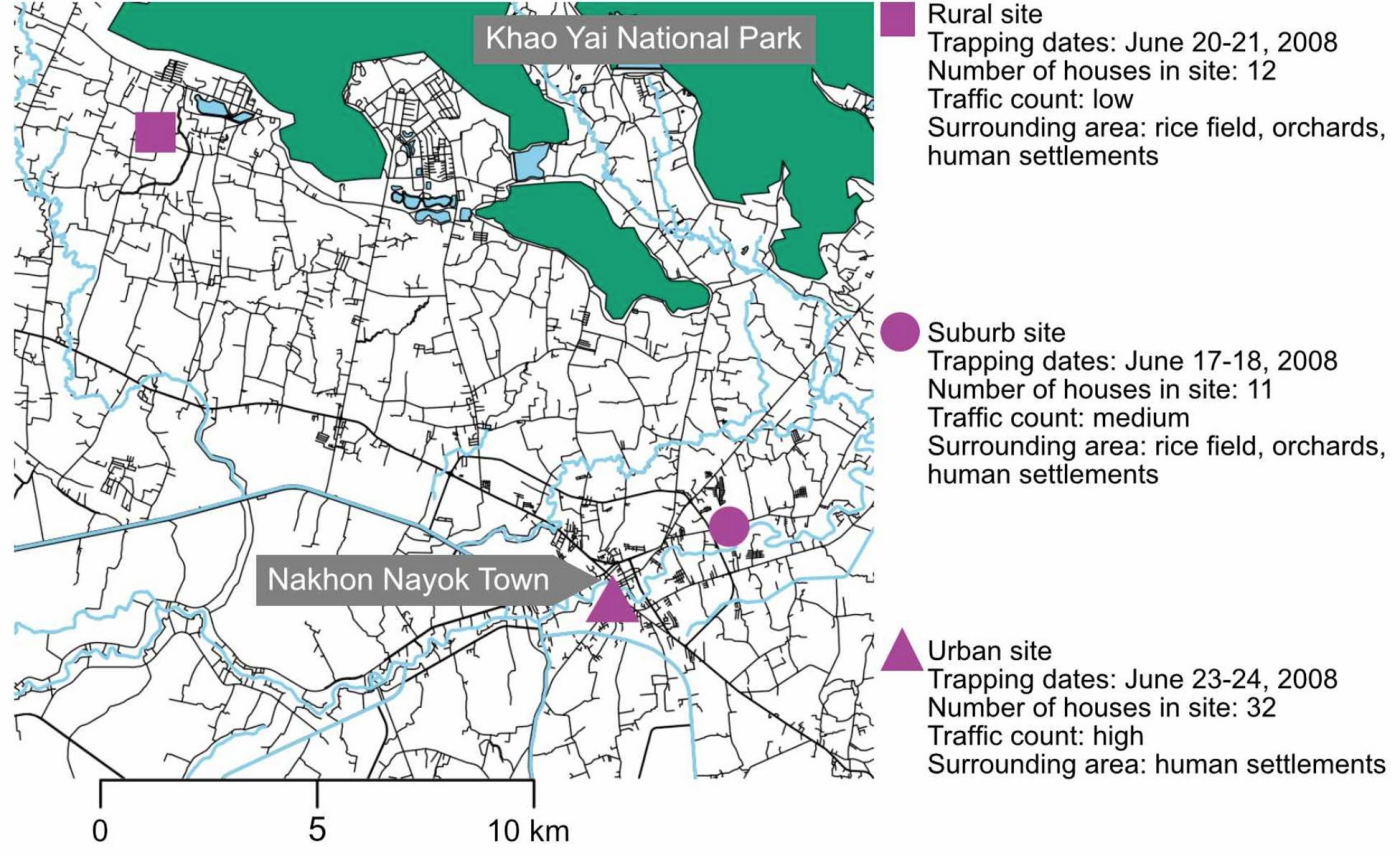
Three study sites located along an urbanization gradient in Nakhon Nayok province of central Thailand. Detailed habitat characteristics can be found in our previous publication^27^. The map was created using R v3.5.3 (www.r-project.org).

In this study, we included nine samples of three vector species: *Ar. subalbatus*, *Cx. fuscocephala*, and *Ma. uniformis,* each collected from rural, suburban, and urban habitats (Fig. 1). For each sample, we combined 25 female mosquitoes of the same species collected from the same study site during the same sampling period. We visually confirmed that these mosquitoes were not blood-engorged. Mosquitoes were then homogenized in 250 μl of Phosphate Buffered Saline using a Tissue Lyser II (QIAGEN, USA) and stainless steel beads before mixing with TRIzol LS (Invitrogen, USA) at the ratio of 1:7. Samples were kept at − 80 °C until RNA extraction according to the manufacturer’s protocol.

### Mosquito specific ribosomal RNA probes, and host ribosomal RNA depletion

In order to increase the relative proportion of microbial RNA, we used biotin-labeled mosquito-specific ribosomal RNA (rRNA) probes to capture and deplete mosquito rRNA prior to sequencing. The procedures for probe construction and rRNA depletion followed a published protocol^28^. In short, the rRNA probes were created by reverse transcribing *Cx. pipiens* (of a laboratory colony) *rRNA* gene using a set of custom designed mosquito-specific small subunit (SSU) and large subunit (LSU) ribosomal RNA primers attached with T7 promoter sequences (MEGAscript T7 Transcription Kit, Invitrogen, USA). The primer sequences are listed in Supplementary Table 1. The biotin-labeled UTP (Roche Life Science, USA) and CTP (Enzo Life Sciences, USA) were used. Since *rRNA* is relatively conserved between these mosquito species (e.g. 81–92% identity for *28 s rRNA*, and 91–97% identity for *18 s rRNA*; based on our preliminary analysis), we surmised that the probes constructed from *Cx. pipiens* would bind to other species of mosquito rRNA.

To deplete mosquito rRNA from the samples, the probes and RNA samples were combined and allowed to hybridize at 70 °C for 5 min, before ramping down to 25 °C with increments of 5 °C and one minute. Next, we used streptavidin magnetic beads (NEB, USA) to separate out the hybridized rRNA and the un-hybridized probes. The bead wash procedures were repeated three times to improve depletion efficiency. The resulting rRNA-depleted RNA samples, along with their paired non-depleted RNA samples, were used for next steps. We designate rRNA depleted sample “RD” and un-depleted control sample “UD” for the rest of this manuscript.

### cDNA synthesis, cDNA library preparation, and high-throughput sequencing

The first strand cDNA synthesis was carried out using SuperScript III Reverse Transcriptase (Life Technologies) and random primers following the manufacturer’s protocol. This was followed by the second strand cDNA synthesis using DNA Polymerase I (New England Biolabs). Sequencing libraries were created using Nextera XT kit (Illumina). Purified libraries were quantified by Qubit 2.0 Fluorometer (Life Technologies, USA) and assessed by Agilent 2100 Bioanalyzer (Agilent Technologies, USA) for average fragment sizes. Bioanalyzer traces for representative paired RD and UD samples are shown in Supplementary Figure 1. The libraries were combined using equimolar ratio and were sequenced on the Illumina MiSeq platform (Illumina, USA) using the paired-end V2 500 cycle reagent kit at the Center for Comparative Genomics, California Academy of Sciences. We performed two separate sequencing runs (Supplementary Table 2). Both RD and UD no-template controls (NTC) were included in each sequencing run.

### Sequence processing and viral contig identification

Raw sequences were filtered for quality and the Illumina adapters were trimmed using Trim Galore! v0.4.5 (https://github.com/FelixKrueger/TrimGalore) using default parameters. The remaining reads were assembled into contigs using Trinity v2.8.4 with default parameters^35,36^. To identify viruses in the sample, the resulting contigs were compared to the NCBI protein database (downloaded September 25, 2018) using DIAMOND v0.9.22^37^ with an E-value cutoff of 10^−03^. The most similar match was identified as having the lowest e-value.

### Viral phylogenetic analysis and coverage estimation

In order to increase the coverage of viral genomes, viral contigs belonging to the same virus across samples and across the two preparation types were assembled using Sequencher v5.1 (Gene Codes Corporation, USA). The resulting assembled genomes or partial genomes were checked manually. The open reading frames (ORF) were predicted using ORFfinder (www.ncbi.nlm.nih.gov/orffinder/) and were confirmed to match viral proteins using NCBI blastp suite. To infer phylogenetic relationships between RNA viruses, selected viral amino acid sequences were aligned using MAFFT v7 employing the E-INS-i algorithm^38,39^. The best-fit model of amino acid substitutions was determined using ProtTest v3.4.2^40^. Phylogenetic analysis was performed using RAXML blackbox^41^ implemented in Cipres Science Gateway application^42^. The resulting phylogenetic trees were visualized in iToL v4.4.1^43^.

The sequence depth and coverage for each viral genome or partial genome was determined by mapping the raw, quality-checked reads to the nucleotide sequences of the viral genome or partial genome using Bowtie2 v2.2.6^44^ and default parameters. Samtools v1.8^45^ was used to process the resulting SAM file from the Bowtie alignment to calculate the average read depth and coverage.

### Identification of endogenous virus elements (EVEs)

Because the genomes of the three mosquito species are currently unavailable, we cannot definitively exclude EVEs from our data. Instead, we manually checked and removed virus contigs closely related to known EVEs using NCBI blastx. For example, contigs similar to multiple sclerosis associated retrovirus were removed^46^.

### Identification of non-viral microorganisms

To classify non-viral reads, Kraken v2.0.7-beta was used^47^. The NCBI’s Nucleotide (nt) database was used to build a Kraken-specific reference database. In order to confirm the genus of the organism, SortmeRNA was used to filter rRNA reads from the data^48^. Then, the rRNA reads were used to search for matches using NCBI blast suite against the nucleotide database with an E-value cutoff of 10^−3^. The most similar match was identified as having the lowest e-value.

### Statistical analysis

To compare virus occurrences across samples, we first normalized the number of reads (i.e. sampling effort) so that they all contained the same number of sequences (n = 418,000, which is the size of the smallest library; Supplementary Table 2). This is done by randomly selecting a subset of raw, quality-checked reads from each sample using Seqtk tool kit (https://github.com/lh3/seqtk). This data set was then assembled de novo using Trinity, searched for viral matches with Diamond, and sequence depths for contigs were determined using Bowtie2 v2.2.6 as described previously. A data matrix containing log-transformed read numbers that mapped to each virus were used as input for principal component analysis (PCA) to assess whether their pattern of occurance is determined by host species or habitat. PCA was performed using the “prcomp” function (R v3.5.3), which performs PCA on the given data matrix.

To compare parameter values such as average length of virus contigs, percentage, and number of reads between RD and UD samples, we used Mann–Whitney U test with the alpha level of 0.05. The non-parametric test was chosen because the data were not normally distributed (as revealed by Q–Q plots and the Shapiro–Wilk test). All statistical analyses, and data visualization were performed using R v3.5.3 and Rstudio v1.2.

### Criteria used for identifying contaminating virus taxa

**Figure 2:**
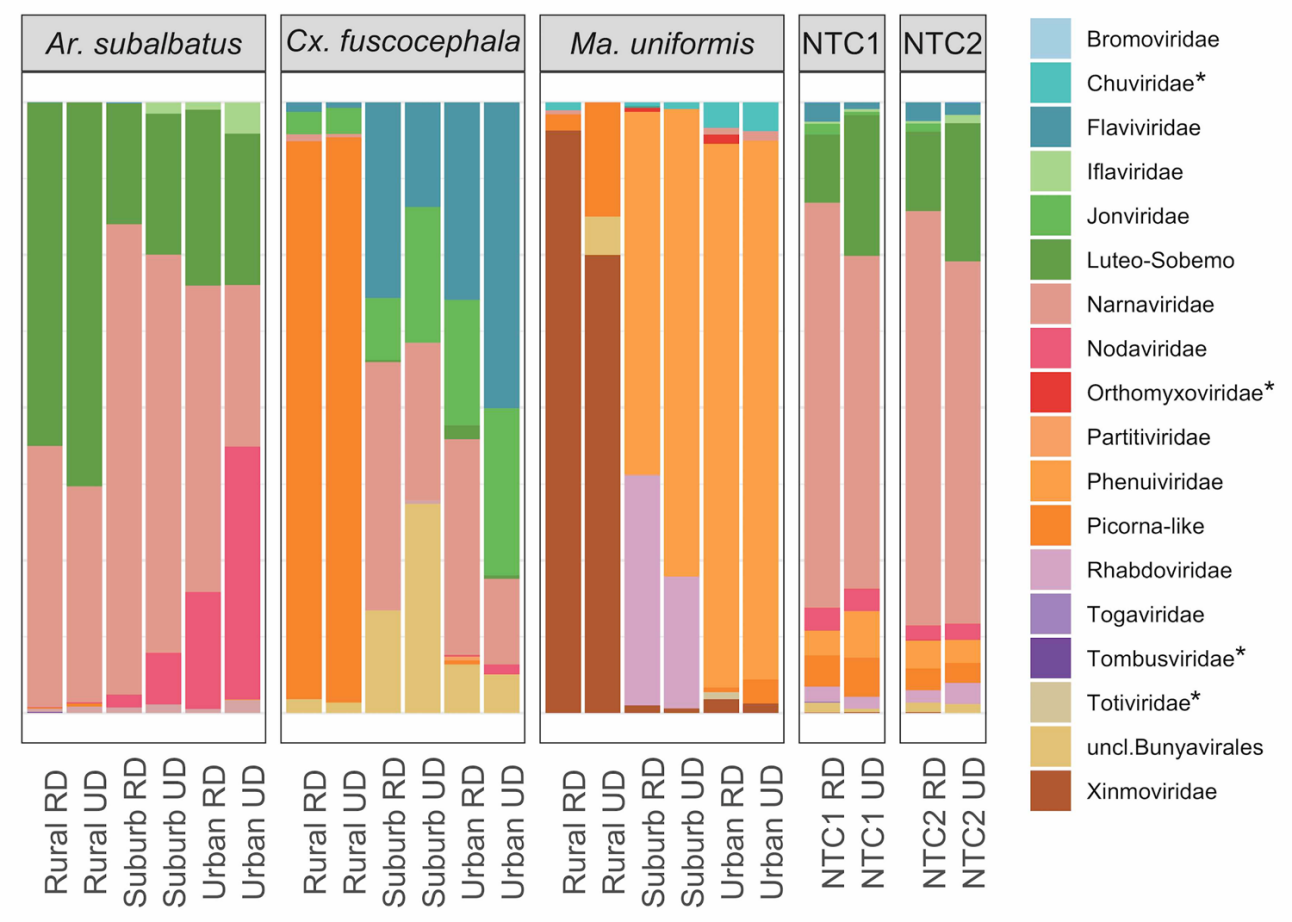
The proportion of virus families found in all mosquito samples and NTCs. RD = rRNA depleted, UD = rRNA not depleted. Asterisks (*) indicate virus groups that are likely contamination. NTC1 and NTC2 refer to no-template controls for separate sequencing runs (Supplementary Table 2).

A given virus taxon was defined as an external contamination if it met *all* of the following: (1) it was found in the no-template controls (NTCs); (2) it was represented by ≤ 80 reads (we chose this cutoff number because a majority, or around 97%, of contigs found in NTCs was represented by 80 reads or less); (3) it was found in mosquito samples in a random pattern (i.e. its presence had no species-specific pattern or habitat-specific pattern); and (4) it is not similar to a previously characterized insect-specific virus. In addition, a given viral taxon was likely a cross contamination between samples if it met criteria (1)–(3) mentioned above. We indicated taxa that are likely contaminants or cross-contaminants in Fig. 2. The complete list of virus contigs found in NTCs is shown in Supplementary Table 3. The complete lists of non-viral taxa found in NTCs according to analysis using SortmeRNA and Kraken are shown in Supplementary Tables 4 and 5, respectively.

## Results

### Read numbers and length

The Illumina MiSeq sequencing generated over 13 million reads. Not including NTCs, the average number of raw reads per sample was 626,988 (SE = 65,936). After quality filtering, the average number of reads dropped to 616,915 (SE = 64,768). Our quality filtering step removed on average 1.53% of raw reads (Supplementary Figure 2). The quality filtered reads were then assembled to produce an average of 5032 contigs per sample (SE = 1216). The average number of assembled contigs per RD sample was significantly higher than in the UD samples (paired t-test, t = 4.2573, *p* value = 0.003; Supplementary Figure 2). The average read length after the filtering step across all samples was 184 bp (SE = 5). The average contig length was 308 bp (SE = 6). The numbers of reads and contigs for all samples are listed in Supplementary Table 2.

### Mosquito viromes

At least 21 putative viruses were identified from all samples. These viruses belong to the order *Bunyavirales* (family *Phenuiviridae*, *Jonviridae*, and an unclassified clade), order *Mononegavirales* (family *Rhabdoviridae* and *Xinmoviridae*), family *Flaviviridae*, family *Iflaviridae*, an unclassified clade closely related to *Luteoviridae* and *Sobemovirus* (referred to as Luteo-Sobemo-related group^6^), unclassified clades related to family *Narnaviridae*, family *Nodaviridae*, and family *Picornaviridae* (Fig. 2). In addition, we recovered a small number of virus reads (≤ 80) aligning to contigs that were classified in the following groups: *Togaviridae*, *Totiviridae*, *Tombusviridae*, *Chuviridae*, *Orthomyxoviridae*, *Partitiviridae*, and an unclassified clade closely related to negeviruses. Notable amongst these was the insect-specific Negevirus-like virus, and the virus in the family *Togaviridae*, which had 98% amino acid similarity to a mosquito-borne zoonotic virus: Getah virus of the genus *Alphavirus*. Detailed information about all viral contigs found in this study, and their most closely related viruses is listed in Supplementary Table 3.

### Virome structure across host species and habitat

**Figure 3:**
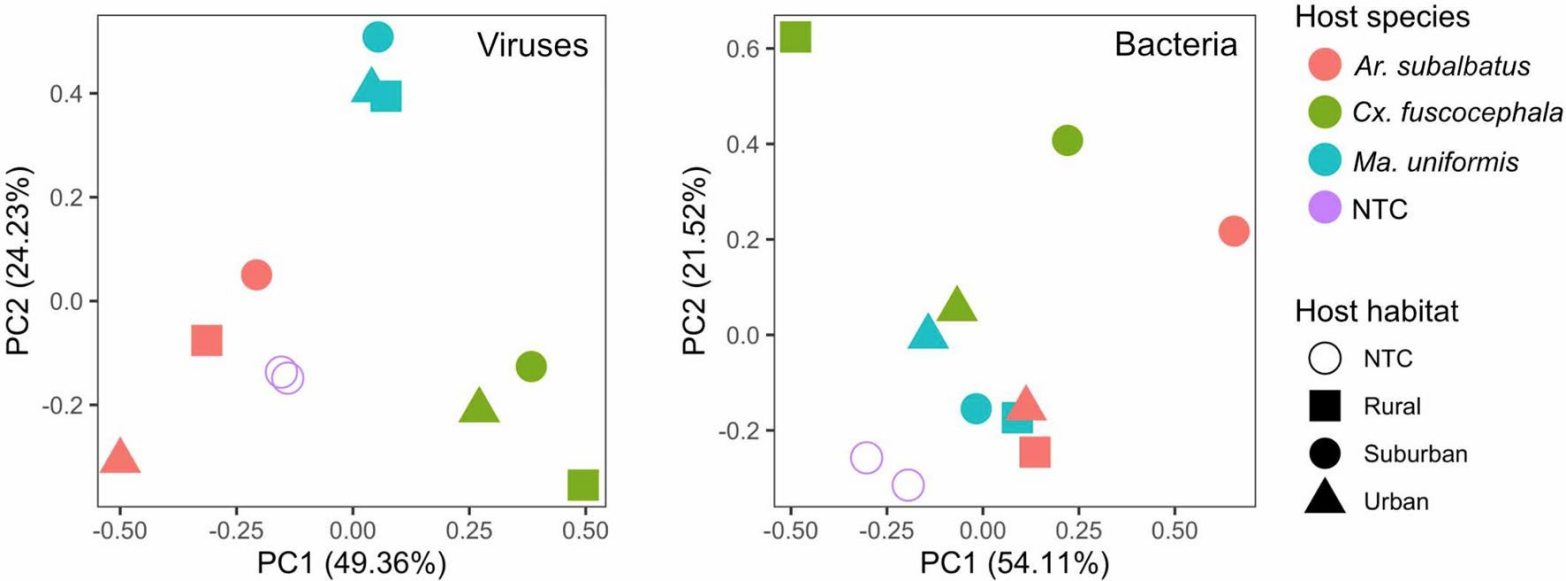
Similarity of virus community (**a**) and bacteria community (**b**) from RD samples. A Principle Component Analysis was performed based on log-transformed read numbers of bacterial and virus taxa found in each mosquito species and habitat type.

We used Principal Component Analysis to determine how mosquito viromes differ across host species relative to habitats. The results indicate that the virus communities in both the RD (Fig. 3a) and UD (Supplementary Figure 3) samples were grouped based on host species rather than the habitat of mosquito collection. This is in contrast to the bacterial community (Fig. 3b), where grouping patterns did not show association with host or habitat type.

### Depth and coverage: comparison between RD and UD samples

We tested a laboratory method to deplete host ribosomal RNA from total RNA. Table 1 shows the comparison between samples with (RD) and without (UD) host rRNA depletion and the statistical tests used. The total number of virus contigs and virus groups was higher in RD than in the UD sample set. The average percentage of virus reads per RD sample was higher than in the UD sample, though this was not statistically significant (n = 18, *p* value = 0.094). The average percentage of viral reads per contig in the RD samples was significantly higher than UD samples (n = 292, *p* value = 0.023). Successful depletion was apparent only for the small subunit of mosquito ribosomal (SSU) gene but not for the large subunit (LSU). The number of reads mapped to the SSU gene was significantly lower in RD samples than in UD samples (*p* value < 0.001), a difference that was not observed for the LSU.

**Table 1:** Comparison of characteristics between rRNA-depleted samples (RD) and undepleted samples (UD).

Characteristics	UD samples	SD	RD samples	SD	n	*p* value	Note
Total number of virus group found	12		18				Group was defined at family level except for "luteo-sobemo" group
Total number of viral contigs found	218		419				Contigs from trinity
Average length of viral contigs	541	566	573	551	637	0.491	Contigs from trinity
Average percentage of virus reads in total reads per sample	0.095	0.160	0.438	0.661	18	0.094	
Average percentage of viral reads in total reads per contig	0.005	0.0003	0.017	0.109	292	0.023	Assembled contigs
**Average percentage of reads mapped to the Large Subunit region of mosquito ribosomal gene (LSU) in total reads per sample**							Reference sequence Accession number X89642
*Cx. fuscocephala* sample*s*	16.213	0.637	10.228	3.863			
*Ar. subalbatus* sample*s*	11.157	1.573	10.296	1.853			
*Ma. uniformis* sample*s*	10.791	2.066	13.487	1.687			
All samples	12.720	2.945	11.337	2.811	18	0.297	
**Average percentage of reads mapped to the Small Subunit region of mosquito ribosomal gene (SSU) in total reads per sample**							Reference sequence Accession number AY988445
*Cx. fuscocephala* sample*s*	38.259	8.177	7.43	1.709			
*Ar. subalbatus* sample*s*	46.079	8.712	8.322	4.484			
*Ma. uniformis* sample*s*	53.734	6.127	12.444	3.230			
All samples	46.024	9.486	9.399	3.706	18	< 0.001	

Statistical analysis was performed using Mann–Whitney U test.

### Evolutionary history

#### Family *Flaviviridae*; Genus *Flavivirus*

**Figure 4:**
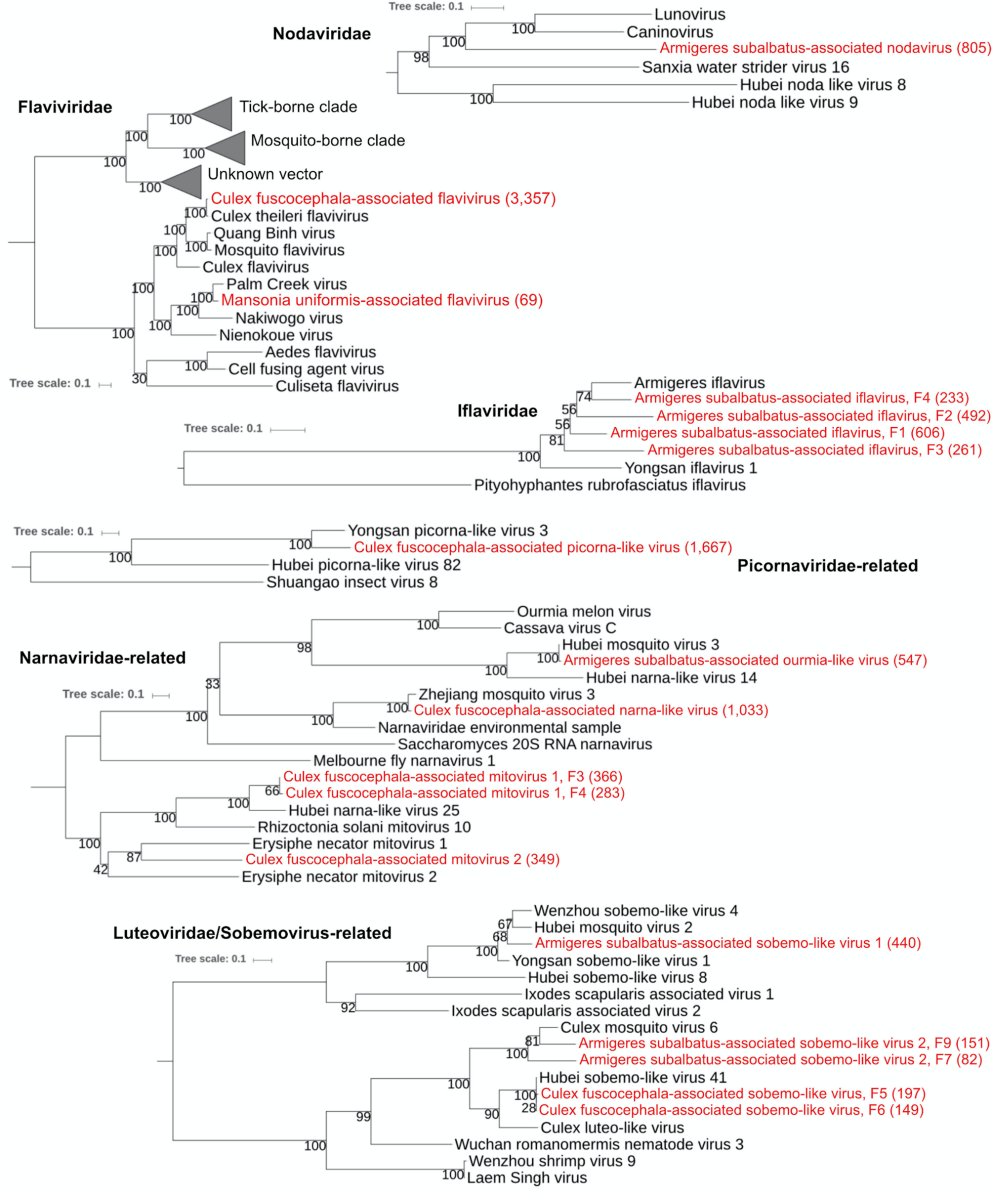
Phylogenetic relationships of the positive sense ssRNA viruses discovered in this study (labeled red) and other known viruses. The analyses were based on RNA-dependent RNA polymerase (RdRp) amino acid sequences, except for Flaviviridae and Iflaviridae trees, in which polyproteins were used. The numbers in parentheses are the lengths of the amino acid sequences.

Viruses of the family *Flaviviridae* possess positive-sense single-stranded RNA (ssRNA) genomes of approximately 9–13 kbp encoding a single polyprotein^49^. We found at least two partial viral genomes from the insect-specific clade of the genus *Flavivirus* in our samples (Fig. 4). The first putative virus, Culex fuscocephala-associated flavivirus, found in all three *Cx. fuscocephala* samples, formed a clade with many flaviviruses isolated from *Culex* spp. We were able to recover > 10 kbp of its genome. The most similar sequence (99% amino acid identity) belonged to a flavivirus isolated from multiple *Culex* species (*Cx. tritaeniorhynchus*, *Cx. vishnui*, or *Cx. fuscocephala*) collected in Myanmar^50^. The second putative virus, Mansonia uniformis-associated flavivirus (Fig. 4), was represented by a short contig (207 bp) found in the suburban *Ma. uniformis* sample. It is related to Palm Creek virus isolated from *Coquillettidia xanthogaster* in Australia (78% amino acid identity), and Nakiwogo virus isolated from *Mansonia* sp. in Uganda (45% amino acid identity).

#### Family *Iflaviridae*; Genus *Iflavirus*

The viruses of the genus *Iflavirus* possess linear positive-sense ssRNA genomes of approximately 9–11 kb in length encoding a single polyprotein^51^. All members infect arthropod hosts with the majority infecting insects^51^. We found four non-overlapping partial genomic fragments of iflavirus, provisionally called Armigeres subalbatus-associated iflavirus (Fig. 4), in the urban and suburban *Ar. subalbatus* samples. Based on our phylogenetic analysis, all four fragments are clustered with Armigeres iflavirus found in an unknown *Armigeres* species collected in the Philippines^52^. The percent amino acid identity to the reference Armigeres iflavirus ranged from 48 to 79% (73–606 amino acids long).

#### Unclassified clade closely related to *Luteoviridae* and *Sobemovirus*

Both *Sobemovirus* and *Luteoviridae* are groups of plant viruses with linear and non-segmented positive sense ssRNA genomes of approximately 4 kb and 6 kb in length, respectively^53^. A recent study found many new virus genomes in diverse invertebrate species classified to a novel clade that is phylogenetically divergent but related to *Sobemovirus* and *Luteoviridae*^6^. At least three partial virus genomes identified in our samples belonged to this novel clade.

Using RNA-dependent RNA polymerase (RdRp) proteins to construct a phylogeny, the putative viruses identified in this study clustered with 3 different clades. The first putative virus, provisionally called Armigeres subalbatus-associated sobemo-like virus 1 (Fig. 4) found in all *Ar. subalbatus* samples fell within a clade containing Yongsan sobemo-like virus 1, Wenzhou sobemo-like virus 4, and Hubei mosquito virus 2. The second putative virus, provisionally called Culex fuscecephala-associated sobemo-like virus (Fig. 4), represented by two non-overlapping RdRp fragments, was similar to Hubei sobemo-like virus 41 (95% and 97% amino acid identity). This virus was found in all *Cx. fuscocephala* samples. The last putative virus, provisionally called Armigeres subalbatus-associated sobemo-like virus 2 (Fig. 4), found in the urban *Ar. subalbatus* sample, formed a clade with Culex mosquito virus 6.

#### *Narnaviridae* and the closely-related Ourmia-like virus

Viruses in the family *Narnaviridae* possess a single molecule of non-encapsidated positive-sense ssRNA of 2.3–3.6 kb^54^. Two genera are currently recognized within this family: genus *Mitovirus* (fungi viruses) and *Narnavirus* (viruses of yeast)^53^. Members of the genus *Ourmiavirus*, closely related to the *Narnaviridae*, are plant viruses with genomes consisting of three positive-sense ssRNAs^55^. At least 4 partial virus genomes could be classified within the *Narnaviridae* and *Ourmiavirus* in our study. The first virus, provisionally called Armigeres subalbatus-associated ourmia-like virus (Fig. 4), was found in large numbers (0.15–0.38% of total reads) in all three *Ar. subalbatus* samples. Its partial genome was similar to the sequence of Hubei mosquito virus 3 (99% amino acid sequence similarity) identified in a pool of multiple mosquito species collected in China^6^. Our newly described ourmia-like virus clustered within a clade of ourmia-like invertebrate viruses.

The second virus, provisionally called Culex fuscocephala-associated narna-like virus (Fig. 4) was similar to the sequence of Zhejiang mosquito virus 3 (93% amino acid identity), which was identified in a pool of multiple mosquito species collected in China^6^. This virus was found in all *Cx. fuscocephala* samples. The third virus, provisionally called Culex fuscocephala-associated mitovirus 1 (Fig. 4), was similar to Hubei narna-like virus 25 (68–69% amino acid identity). This virus was found in the rural *Cx. Fuscocephala* sample. The fourth virus, provisionally called Culex fuscocephala-associated mitovirus 2 (Fig. 4) was similar to Erysiphe Necator mitovirus 1 (36% amino acid identity). This virus was also found in the rural *Cx. fuscocephala* sample.

#### Nodaviridae

The family *Nodaviridae* includes two genera, *Alphanodavirus* (infects insects) and *Betanodavirus* (infects fish)^53^. Genus *Gammanodavirus* infecting prawn and shrimp has also been proposed^56^. Nodaviruses genomes consist of two molecules of positive sense ssRNA. RNA-1 (3.1 kb) encodes protein A with functions such as polymerase, and RNA-2 (1.4 kb) encodes protein alpha^57^. Phylogenetic analysis showed that the partial RNA-1 found in our study (~ 2.5 kb) formed a clade with Lunovirus (from a carnivore fecal virome study), Caninovirus (from a canid gut virome study), and Sanxia water strider virus 16 (from a pool of multiple water strider species collected in China). The most similar sequence was Sanxia water strider virus 16 (39% amino acid similarity). This virus, provisionally called Armigeres subalbatus-associated nodavirus (Fig. 4), was found in all three *Ar. subalbatus* pools. Another contig resembling capsid protein (~ 1 kb), likely representing the RNA-2 fragment of the same virus, was also found in all *Ar. subalbatus* samples.

#### Unclassified clade closely related to *Picornaviridae*

Sequences from a virus closely related to the family *Picornaviridae* was discovered in high numbers in the rural *Cx. fuscocephala* sample (1.89% of total reads). The assembled partial genome of this virus, provisionally called Culex fuscocephala-associated picorna-like virus (Fig. 4), was most similar to the genome of Yongsan picorna-like virus 3 (70% amino acid identity) found in *Aedes vexans* from South Korea^58^. *Picornaviridae* is a large and diverse family containing viruses with positive sense ssRNA genomes ranging from 6.7 to 10.1 kb^58^. Recent metagenomic studies have found a great number of novel picorna-like viruses in invertebrates including mosquitoes^6,14^. It has been suggested only recently that a new family, *Polycipiviridae*, assigned to the order *Picornavirales*, should be adopted to include insect-associated picorna-like viruses^59^.

#### Order *Bunyavirales*

**Figure 5:**
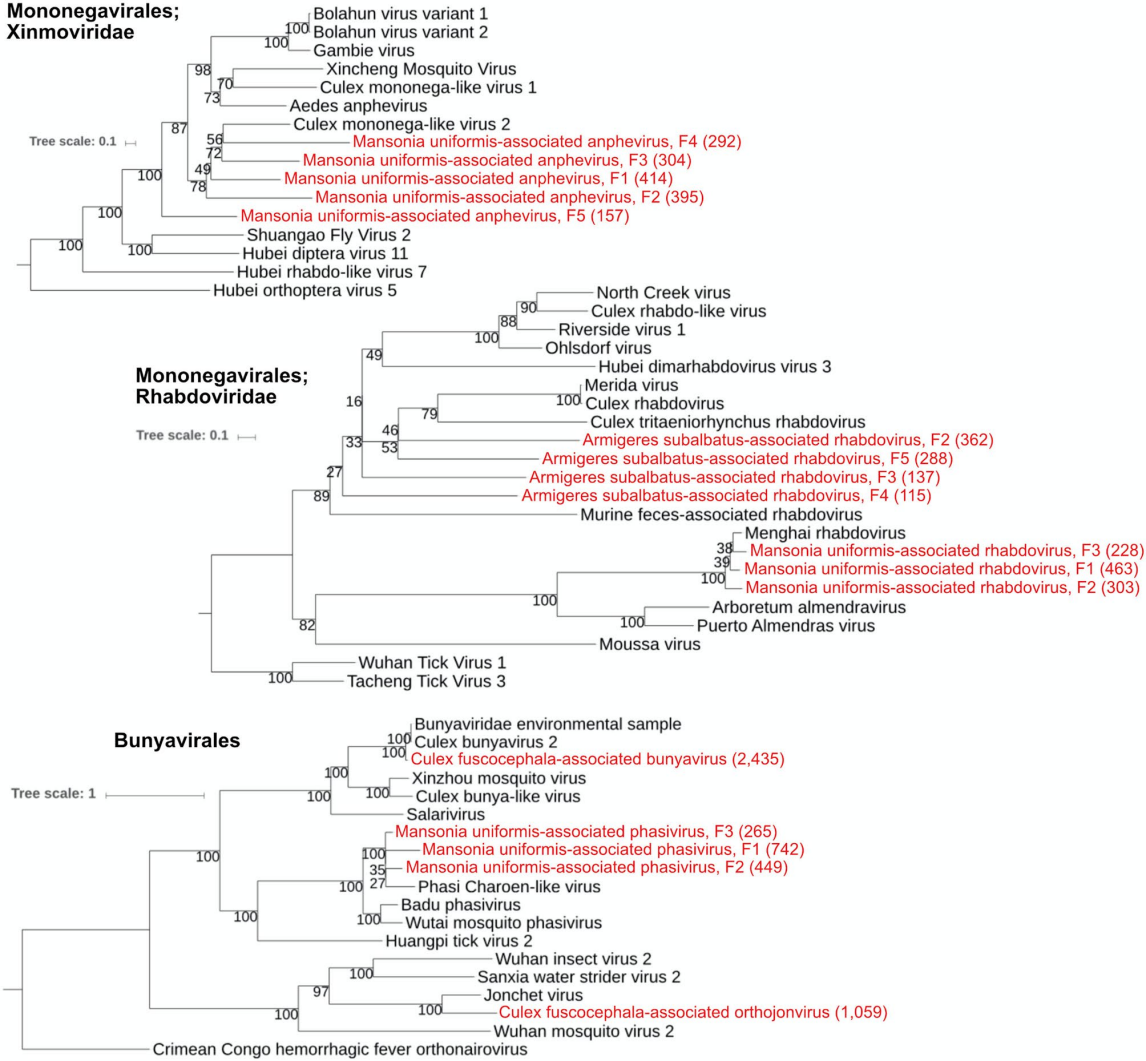
Phylogenetic relationships of the negative sense ssRNA viruses discovered in this study (labeled red) and other known viruses. The analyses were based on RNA-dependent RNA polymerase (RdRp) amino acid sequences. The numbers in parentheses are the lengths of the amino acid sequences.

Order *Bunyavirales* includes viruses with segmented, negative-sense or ambisense single-stranded RNA (ssRNA) genomes distributed among 9 families (International Committee on Taxonomy of Virus, ICTV; Taxonomic Proposal 2016.030a-vM). The genomes consist of two to six segments encoding structural proteins, and one or more non-structural proteins^60^. Sequence analysis of the RdRps indicated that the *Bunyavirales* contigs in our samples could be assembled into at least three distinct clades (Fig. 5). The first putative virus, provisionally called Culex fuscocephala-associated bunyavirus (Fig. 5), found in all *Cx. fuscocephala* samples, fell in an unclassified clade. This virus was similar to those found in *Cx. pipiens* collected in California (~ 93% amino acid identity)^8,12^. The second putative virus belongs to the genus *Phasivirus* in the family *Phenuiviridae* (Fig. 5). This putative virus, provisionally called Mansonia uniformis-associated phasivirus, was found in the suburb and urban *Ma. uniformis* samples. It was similar to Phasi Charoen-like phasivirus found in *Ae. aegypti* in Thailand and China (amino acid identity varies from 58 to 85% depends on the fragment)^13^. The third putative virus, provisionally called Culex fuscocephala-associated orthojonvirus (Fig. 5), was found in all three *Cx. fuscocephala* samples. The closest relative (32–53% amino acid identity) was the Jonchet virus (family *Jonviridae*) documented from mosquitoes in Côte d’Ivoire^61^.

#### Order *Mononegavirales*: *Rhabdoviridae* and *Xinmoviridae*

The family *Rhabdoviridae* includes viruses with genomes of 10.8–16.1 kb, usually with 5 genes encoding structural proteins^62^. The family is ecologically diverse with members infecting plants, fish, reptiles, birds, and mammals as well as arthropod hosts or vectors^62^. Our phylogenetic analysis of the L proteins (RdRp) suggested that there were at least two putative rhabdoviruses in our mosquito samples. The first virus, provisionally called Mansonia uniformis-associated rhabdovirus (Fig. 5), was found only in the suburban *Ma. uniformis*. This virus formed a clade with Menghai rhabdovirus (77–96% amino acid similarity), which was recently isolated from *Ae. albopictus* from China^63^. Another set of rhabdovirus L gene fragments was recovered in all three *Ar. subalbatus* pools (Armigeres subalbatus-associated rhabdovirus). These fragments showed similarity to known virus sequences (33–50% amino acid similarity) and the closest similarity was to Culex rhabdovirus. Because of their small sizes, the phylogenetic analysis of these L fragments did not result in a strongly supported clade and we cannot conclude whether these fragments belong to the same virus (Fig. 5).

We also recovered contigs similar to a virus in the newly proposed genus *Anphevirus* of the family *Xinmoviridae* (ICTV report 2017.016 M.A.v3), which consists of multiple insect viruses. The assembled genomic fragments found in our study (Fig. 5) were closely related to *Culex* mononega-like virus 2 (amino acid sequence similarity ranges from 33 to 50%). This virus, provisionally called Mansonia uniformis-associated anphevirus (Fig. 5), was found mostly in the rural *Ma. Uniformis* sample.

### Phylogenetic relationship of viruses across study sites

In order to determine genetic relationships within those viruses that occurred across study sites, the partial genomes of each virus found in all three sites were subjected to phylogenetic analysis where genome coverage was sufficient. Of the three viruses that were found in high numbers in all sites, we document limited genetic divergence across sites of collection (Supplementary Figure 4).

### Non-viral reads

**Figure 6:**
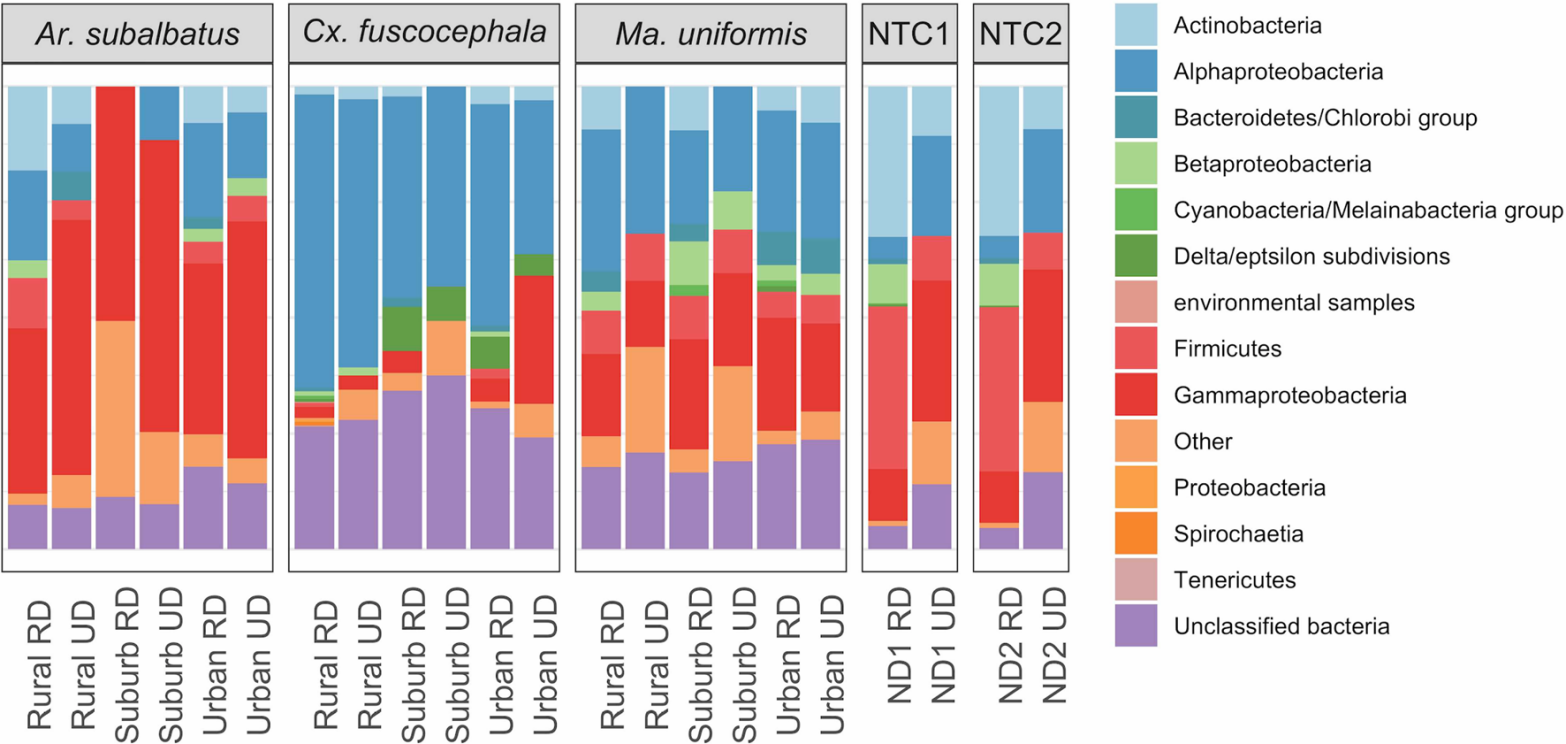
The proportion of bacteria groups found in all mosquito samples and NTCs. RD = rRNA depleted, UD = rRNA not depleted. NTC1 and NTC2 refer to no-template controls for separate sequencing runs (Supplementary Table 2).

The majority of non-viral reads, in both the RD and UD samples, were classified to phylum Arthropoda, averaging 79.80% and 81.08%, respectively (Supplementary Table 5). The average number of reads that could not be classified across all samples was 7.17% and 2.73% for RD and UD samples, respectively. The average numbers of non-virus reads classified to the Bacteria domain were 2.23% and 0.48% for RD and UD samples, respectively. The proportion of bacteria groups in all samples is shown in Fig. 6. The dominant bacterial group differed in each mosquito host species: in *Ar. subalbatus* the majority were in the class Gammaproteobacteria (average = 48.73% of all bacterial reads) while the majority of bacteria found in *Cx. fuscocephala* were in the class Alphaproteobacteria (48.19%). The composition of the bacteria community in *Ma. uniformis* samples was more evenly distributed, dividedly between Alphaproteopbacteria (26.12%) and Gammaproteobacteria (19.87%). Other groups of bacteria found in mosquito samples included: phylum Actinobacteria, averaging 3.58% of all bacterial reads across all samples; phylum Firmicutes, 2.39%; class Betaproteobacteria, 1.68%; Bacteroidetes-Chlorobi group, 1.54%; and Delta-Epsilon Proteobacteria subdivision, 1.53%.

In terms of individual bacteria of note, *Wolbachia* (Alphaproteobacteria), a genus of common obligate intracellular bacteria found in a wide range of invertebrate taxa^64^, was found in all samples as indicated by 16 s rRNA contigs recovered. We also found rRNA reads of bacteria relating to common house flies and bee gut-associated *Apibacter* bacteria in all *Cx. fuscocephala* samples, but not other mosquito species (Supplementary Figure 5). Also restricted to the three *Cx. fuscocephala* samples were bacteria similar to *Helicobacter sp.* of the Epsilon-proteobacteria, and bacteria similar to the bee-associated *Frischella sp.* and *Gilliamella sp.* of the Gammaproteobacteria.

Multiple groups of fungi were detected (Supplementary Table 5), averaging 0.56% and 0.21% of all non-viral reads in RD and UD samples, respectively. Common divisions of fungal reads found in all samples were Ascomycota and Basidiomycota. The highest percentage of fungal reads was from the pool of *Cx. fuscocephala* collected from the rural habitat. The majority of fungi in this sample were classified into phylum Zoopagomycota (division Zygomycota), at 2.56% and 0.88% or all in RD sample and UD sample, respectively.

Other organisms within our samples included trypanosomatids, alveolates, plants, algae, platyhelminthes, nematodes, annelids, mollusks, and vertebrates (Supplementary Table 4). Initial analysis of ribosomal RNA reads suggested there existed multiple poorly known species of mosquito-associated eukaryotic microorganisms in our samples. For example, partial trypanosomatid rRNA reads, related to trypanosomatids of multiple insects, were found in all three mosquito species. In addition, partial 18 s rRNA reads similar to *Paratrypanosoma confusum*, a trypanosomatid species recently found in *Cx. pipiens* guts^65^, were detected in *Cx. fuscocephala* samples.

Multiple samples collected from rural and urban sites harbored 18 s rRNA reads similar to nematode species in the Mermithidae, and Setariidae families. Of note is a partial 28 s rRNA read (274 bp) found in the rural *Ma. uniformis* sample that is 100% similar to *Setaria digitata*, a mosquito-borne zoonotic nematode species infecting ungulates in Asia^66^. *Ma. uniformis* sample collected in suburban site harbored reads likely belong to *Brugia malayi* (99–100% similarity, 215 and 431 bp).

Several rRNA reads were recovered in the urban *Cx. fuscocephala* sample related to trematode of the order Plagiorchiida such as *Collyriclum faba*, *Paralechithodendrium longiforme*, and *Lecithodendrium linstowi*. *C. faba* is a bird parasite that has aquatic gastropods as the first intermediate host and mayflies as the second intermediate host^67^. On the other hand, *P. longiforme* and *L. linstowi* are bat parasites with unknown second intermediate hosts^68,69^.

Lastly, 18 s rRNA reads of *Ascogregarina sp.*, a mosquito-specific apicomplexan parasite, were found in rural and urban *Ar. subalbatus* samples and were most similar to *A. armigerei* (99.4%, 1061 bp). The genus *Ascogregarina* parasitizes mosquitoes and sandflies, and is relatively host specific: *A. taiwanensis* infects *Ae. albopictus*, *A. culicis* infects *Ae. aegypti*, and *A. armigerei* infects *Ar. subalbatus*^70,71^.

## Discussion

In this study, we used a high throughput RNA sequencing metagenomic approach to characterize the mosquito-associated virome and microbiome. We identified at least 21 putative RNA viruses in three vector species: *Ar. subalbatus*, *Cx. fuscocephala*, and *Ma. uniformis*. We chose the three mosquito species because of their abundance and potential vector status in central Thailand^27^. *Ar. subalbatus* transmits Japanese encephalitis virus, *Wuchereria bancrofti*, and dog heartworm *Dirofilaria immitis*^72^. Their larvae have been found in nutrient enriched water including septic tanks and polluted stagnant water^73^ as well as less enriched bamboo stumps, artificial containers, and tree holes^74^. *Cx. fuscocephala*, often associated with flooded rice field and agricultural lands^75–77^, is among multiple *Culex* mosquito species that transmit Japanese encephalitis^78,79^. *Mansonia uniformis* is a vector of *Wuchereria malayi.* They are associated with vegetated water habitat such as ponds and canals with floating vegetation, rice fields, and swamp forest^80–82^.

According to the Principle Component Analysis, we found that the pattern of viruses in our mosquito samples was defined primarily by the host species rather than by geographical location. Viruses are obligate intracellular parasites that can only function when inside host cells, necessitating specific host cell recognition, entry, and manipulation of multiple host cell molecular mechanisms in order to complete their replication cycles. These properties limit viruses’ biological host ranges. It is not surprising that we observe this host species-specific rather than location-specific pattern. This is in contrast to the bacterial community, where grouping patterns did not show association with host or location. Even though the full factorial design in our study allowed us to investigate the relative importance of ecological factors in structuring mosquito virome, the small sample size limits our ability to generalize this finding.

On the other hand, the same virus species found in the same mosquito species but from different collection sites showed limited genetic divergence (Supplementary Figure 4), suggesting that the viruses are shared between mosquito populations, at least within the geographical scale of this study (the three study sites are within 20 km apart). Other studies comparing mosquito-specific RNA viruses isolated from wider geographical areas found that they showed genetic similarity across continents^83,84^. The results from our study lead to important implications for the surveillance of emerging viral diseases: in order to capture as much viral diversity as possible, monitoring efforts should sample a wide diversity of mosquito species, rather than focus on a narrow set of mosquito species albeit over a larger geographic landscape.

We also tested a host rRNA depletion method which could reduce the cost of sequencing without the need to pool too many individuals into a single sample. Our analysis indicated that we successfully depleted rRNA of the mosquito’s SSU but not of the LSU. Even then, the host rRNA depletion as adopted in our study significantly increased the percentage of virus reads in the sample as well as the coverage for virus contigs. However, our caution is that depletion was time consuming such that the same gains in virus detection could have been achieved for less cost in time with an additional sequencing run, especially when the cost of sequencing may continue to come down in the future.

We found multiple putative viruses associated with certain mosquito species, suggesting that they are likely mosquito-specific. For example, Armigeres subalbatus-associated ourmia-like virus was classified to a novel insect virus clade closely related to plant viruses in the genus *Ourmiavirus*. It was found in high numbers in all *Ar. subalbatus* samples but not in the other two mosquito species. A recent NGS-based study^6^ found multiple invertebrate viruses in well-defined clades distinct but related to both *Ourmiavirus* and *Narnavirus*^55^. According to a recently published ICTV report, there could be a future establishment of a new virus family “*Ourmiaviridae*”^55^. The family would comprise a genus for ourmia-like mycoviruses, an existing genus containing plant viruses, and a genus for ourmia-like viruses isolated from invertebrates (which may contain the ourmia-like virus in our sample)^55^. Another putative virus, Culex fuscocephala-associated narna-like virus, was found in *Cx. fuscocephala* and not the other two mosquito species. This putative virus formed a monophyletic group with a virus found in *Cx. pipiens*^12^ and other arthropods^6^. Although this group of viruses was closely related to mycoviruses, it likely contains a group of novel insect-specific viruses.

Another insect-specific virus, Ma. uniformis-associated anphevirus, was recovered mostly in the rural sample of *Ma. uniformis,* suggestive of habitat restriction. Interestingly, the newly proposed genus *Anphevirus* consists of multiple insect viruses including those isolated from *Culex*^85^, *Anopheles*^5^, and *Aedes* mosquitoes^84^*.* A recent study showed that Aedes anphevirus may reduce DENV replication in cell culture^84^. Our phylogenetic analysis indicated that Ma. uniformis-associated anphevirus is separated from other mosquito anpheviruses (Fig. 4). Thus, we speculate that Ma. uniformis-associated anphevirus could be a *Mansonia*-specific clade*.*

It is important to note that the virus names given in our manuscript are only provisional and not species names. The ICTV sets different species demarcation criteria that vary depending on virus groups; and the criteria often include information other than virus genetic sequences. Lacking relevant information for species assignment, we cannot classify the viruses found in our study into the same species of existing virus, nor can we establish a new species. Ideally, future studies should be done to assign each of these viruses to species.

Our non-targeted sequencing approach reveals multiple interesting mosquito-associated non-viral microorganisms such as a Bacteroidetes species (found in *Cx. fuscocephala* samples; Supplementary Figure 5) similar to the bee-associated *Apibacter sp.*, and a not yet described digenean trematode species in which mosquitoes may serve as the second intermediate host (Supplementary Figure 6). The identification of these non-viral organisms is important in their own rights but it can also give further insight into potential hosts of viruses that might be found in the same mosquito pools. Even though the viruses were discovered in mosquitoes, they may infect other mosquito endosymbionts such as bacteria, fungi, and parasites^86^, or ingested organic material such as plants and algae.

We have included no-template controls (NTCs) that were processed alongside mosquito samples from extraction to sequencing. Due to the high sensitivity of NGS and the well-documented risk of contamination in microbiome studies, control samples should always be included to assess contamination^87,88^ that can originate from the environment (e.g. reagents and kits, plastic consumables, dust, and humans), and cross-contamination between samples^87^, occurring either during sample processing (e.g. aerosol contamination, barcode cross-contamination), or sequencing (e.g. barcode sequencing errors and ‘index hopping’)^87^. Despite its importance, not all NGS-based metagenomic studies include controls.

We detected reads belonging to multiple organisms in the NTCs. The proportion of reads in NTCs classified to each taxon is shown in Supplementary Table 5. We observed that abundant taxa were more likely to be recovered in the NTCs. Thus, we suspect limited cross-contamination between samples. Because NTCs have no starting material, even trace amounts of contaminated DNA could be picked up by NGS. On the contrary, mosquito samples contain large amounts of template DNA reads, and only a few contaminated reads would be sequenced. Other types of controls, such as mock community controls should be adopted in future studies along with NTCs. This type of control not only allows a more realistically detection of contamination, but also the quantification of sequencing error and other bias introduced during the library preparation processes^89^.

Endogenous viral elements (EVEs) are defined as viral DNA sequences deriving from retroviruses, DNA viruses, or RNA viruses that present within the genomes of non-viral organisms, including mosquitoes^90^. Unfortunately, we cannot definitively determine whether the contigs identified in this study originate from viruses or EVEs because the genomes of the three mosquito species are currently unavailable, and because EVEs possess non-specific characteristics that cannot be used for their identification. However, we expect that the RNA extraction protocol adopted in our study likely limited the inclusion of EVEs, and the potentially expressed small EVEs (e.g. EVE-derived piRNAs) in our purified RNA samples.

Mosquito-borne disease emergence is thought to be associated with socio-economic and ecological factors arising from urbanization and poverty. In this study, we characterized mosquito-associated viromes and microbiomes across a landscape gradient transformed by human activities and urbanization. In addition, the three vector species differ in their biology and ecology. We found that the mosquito-associated virome component was defined primarily by the host species rather than by the geographical locations or habitats, and multiple viruses spread between sample sites and mosquito populations at the scale of this study. Similar to *Wolbachia*, we know that elements of the vector virome and microbiome can impact vector fitness and competence: we recovered a virus closely related to Aedes anphevirus previously reported to reduce vector competence to dengue^84^. Thus, this study may help inform future use of insect-specific virus (ISVs) in the control of vectors and diseases. Because we found that mosquito species determine virome composition more than habitat, urbanization is likely to have the greatest influence on the distribution of microbes by facilitating invasive vector species. This finding is supported by our previous study indicating that under urbanization mosquito communities become less diverse and skewed towards invasive mosquito species^27^. To test the influence of habitat alone on mosquito-associated virome, future studies should focus on one mosquito species at a time, and sample individually and more intensively across landscape types.

## Supplementary Information

Supplementary Information 1.

Supplementary Information 2.

Supplementary Information 3.

Supplementary Information 4.

Supplementary Information 5.

Supplementary Information 6.

Supplementary Information 7.

Supplementary Information 8.

Supplementary Information 9.

Supplementary Information 10.

Supplementary Information 11.

## Data Availability

The raw sequence reads generated in this study are available at the NCBI Sequence Read Archive (SRA) database under BioProject PRJNA716099; BioSamples SAMN18394705-SAMN18394726. All virus contigs and some 16s and 18s rRNA contigs generated in this study have been deposited in GenBank under accession numbers: MW854367-MW854382, MW858073-MW858116, MW874579-MW874588, and MW879746-MW879751.
