# Supplementary material for: Metagenomic shotgun sequencing reveals host species as an important driver of virome composition in mosquitoes
Source: Sci Rep. 2021 Apr 19;11:8448. doi: 10.1038/s41598-021-87122-0 (PMC8055903; doi:10.1038/s41598-021-87122-0)

**Supplementary Data**

**Article title**

Metagenomic shotgun sequencing reveals host species as an important driver of virome composition in mosquitoes

Panpim Thongsripong, James Angus Chandler, Pattamaporn Kittayapong,

Bruce A Wilcox, Durrell D Kapan, and Shannon N Bennett

**Supplementary Figure 1** Bioanalyzer traces for representative paired RD and UD urban samples


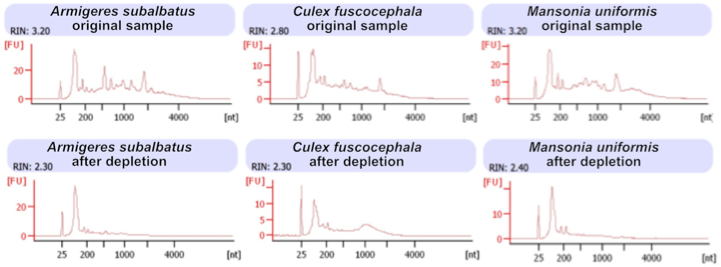

Supplement: Supplementary file 1 — Supplementary Information 1. [file 41598_2021_87122_MOESM1_ESM.docx]
