# Supplementary material for: Metagenomic shotgun sequencing reveals host species as an important driver of virome composition in mosquitoes
Source: Sci Rep. 2021 Apr 19;11:8448. doi: 10.1038/s41598-021-87122-0 (PMC8055903; doi:10.1038/s41598-021-87122-0)

**Supplementary Figure 2.** Boxplots showing average numbers of reads for all samples before and after quality control steps (left), and average numbers of assembled contigs for RD and UD samples (right).


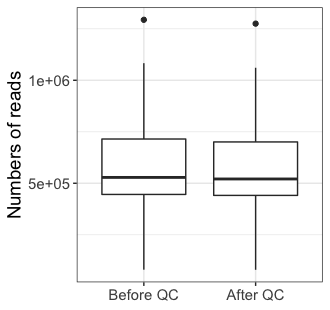

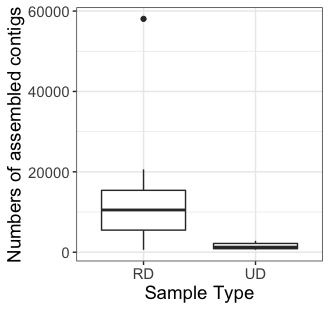

Supplement: Supplementary file 2 — Supplementary Information 2. [file 41598_2021_87122_MOESM2_ESM.docx]
