# Supplementary material for: Metagenomic shotgun sequencing reveals host species as an important driver of virome composition in mosquitoes
Source: Sci Rep. 2021 Apr 19;11:8448. doi: 10.1038/s41598-021-87122-0 (PMC8055903; doi:10.1038/s41598-021-87122-0)
Supplement: Supplementary file 5 — Supplementary Information 5. [file 41598_2021_87122_MOESM5_ESM.docx]

**Supplementary Figure 5.** We found a Bacteroidetes species, similar to the bee gut-associated *Apibacter sp.*, in *Cx. fuscocephala* samples. *Apibacter spp.* were recently isolated from bee guts and are thought to be facultatively associated to multiple bee species [1, 2]. Initial phylogenetic analysis of partial 16s rRNA sequences suggested that the bacteria found in our *Cx. fuscocephala* samples clustered within the same clade as, but separated from, the bee-associated *Apibacter spp.* and another group of similar Bacteroidetes found in termites. Their ribosomal RNAs can be found only in *Cx. fuscocephala* samples collected from all three sites, but not in the other two mosquito species, suggesting that this species specifically associated to the *Culex sp*.

**
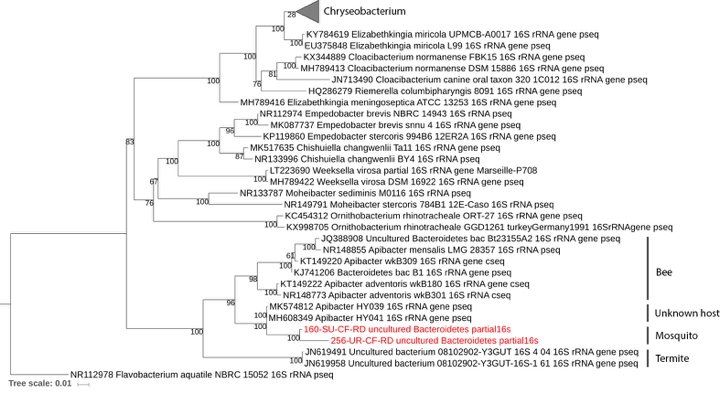
**

1. Kwong WK, Moran NA. *Apibacter adventoris* gen. nov., sp. nov., a member of the phylum Bacteroidetes isolated from honey bees. *Int J Syst Evol Microbiol*. 2016;66: 1323–1329. doi:10.1099/ijsem.0.000882

2. Praet J, Aerts M, Brandt ED, Meeus I, Smagghe G, Vandamme P. *Apibacter mensalis* sp. nov.: a rare member of the bumblebee gut microbiota. *Int J Syst Evol Microbiol*. 2016;66: 1645–1651. doi:10.1099/ijsem.0.000921
