# Supplementary material for: Metagenomic shotgun sequencing reveals host species as an important driver of virome composition in mosquitoes
Source: Sci Rep. 2021 Apr 19;11:8448. doi: 10.1038/s41598-021-87122-0 (PMC8055903; doi:10.1038/s41598-021-87122-0)
Supplement: Supplementary file 6 — Supplementary Information 6. [file 41598_2021_87122_MOESM6_ESM.docx]

**Supplementary Figure 6.** We found ribosomal RNA of a digenean trematode in this study. Initial analysis suggested that this trematode species is related to *Lecithodendrium linstowi*, one of the most prevalent and abundant trematodes of bats [1]. The first intermediate mollusk host of *L. linstowi* was recently identified but the second intermediate insect host remains unknown [1]. The presence of a digenean trematode species similar to *L. linstowi* in *Cx. fuscocephala* could point to the possibility that culicines may represent the missing link in the life cycle of these digenean trematodes.

**
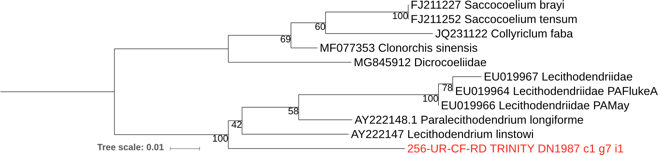
**

[1] Enabulele EE, Lawton SP, Walker AJ, Kirk RS. Molecular and morphological characterization of the cercariae of *Lecithodendrium linstowi* (Dollfus, 1931), a trematode of bats, and incrimination of the first intermediate snail host, *Radix balthica*. Parasitology. 2018;145: 307–312. doi:10.1017/S0031182017001640
